# Council tenancies and hoarding behaviours: A study with a large social landlord in England

**DOI:** 10.1111/hsc.13779

**Published:** 2022-03-20

**Authors:** Bryony Porter, Sarah Hanson

**Affiliations:** ^1^ School of Health Sciences, The University of East Anglia Norwich UK

**Keywords:** community health, harm reduction, hoarding, housing, housing officers

## Abstract

Hoarding behaviours are highly stigmatised and often hidden. People with problematic hoarding behaviours have a higher rate of mental health and other healthcare and social services utilisation. Hoarding is a community health problem, one factor being housing insecurity. Hoarding behaviours represent significant burden to housing providers, impact the community and dealing with it involves multiple community agencies. This study with a city council in England with a large housing stock (over 14,000 properties) in summer 2021 sought to understand the nature, circumstances and extent that hoarding presents. We developed a reporting system and conducted 11 interviews with housing officers in which they described a case to explain their involvement. Our report details the nature of 38 people who hoard: 47% had a known disability or vulnerability, 34% presented a fire and environmental risk, 87% lived alone and 60% were resident in flats. Our qualitative themes are: Working with others, Balancing an enforcement approach, Feeling conflicted, Complex needs of people who hoard and Staff needs. The cases described by the housing officers are combined into six case studies and illustrate the complex, multi‐agency circumstances around decision making and risk stratification. Our findings point to housing officers as frontline professionals dealing with a public health and social care issue which is often the manifestation of complex life histories and mental health conditions. We suggest a greater focus on risk stratification and a more holistic approach to hoarding cases to effectively deal with this most complex of community health and social care issues.

**What is known?**

- When hoarding becomes known to statutory authorities it represents a time‐consuming, multi‐agency social and health problem.
- Dealing with the results of hoarding can be traumatising for the person who hoards and the hoarding behaviours usually re‐occur.

**What this paper adds?**

- When working with people that hoard, housing officers are conflicted in balancing the autonomy of an individual to live in their home in a way that they choose, with risks to the property and the health and safety of the public.
- Housing officers often manage people with complex health, mental health and social needs which extend beyond the boundaries of their role, without access to the necessary expertise to best support people with hoarding behaviours.
- Due to the highly complex nature of hoarding issues, trauma informed approaches might be helpful to the person who hoards and those working with them.

## 1 INTRODUCTION

Hoarding disorder (HD) is defined in the DSM‐5 as a persistent difficulty discarding possessions, resulting in an accumulation of belongings causing severe clutter and the obstruction and congestion of living areas that creates significant distress and impairment in functioning (American Psychiatric Association, 2013). Prevalence is estimated at between 1.5% and 6% of the adult population (Nordsletten et al., 2013; Postlethwaite et al., 2019; Samuels et al., 2008; Timpano et al., 2011) in developed countries. However, estimates of hoarding and the subsequent management of people who hoard are limited due to hoarding behaviour being hidden; associated with embarrassment and shame; hoarding being highly stigmatised; the strong emotional attachments people have to possessions and issues around defensiveness and lack of insight (Chasson et al., 2018; Frost et al., 2010; Kellett & Knight, 2003). The risk factors and possible causes of hoarding are not entirely understood; it may well be due to a combination of factors (Dozier & Ayers, 2017); one study noted that 76% of their sample (*n* = 751 self‐reported people who hoard) had a history of interpersonal violence (Tolin et al., 2010) and there is evidence that it may be correlated with previous traumatic and stressful life events (Hombali et al., 2019; Tolin et al., 2010).

An early study to examine the economic and social burden of self‐reported people who hoard found that they were more likely to have a higher rate of healthcare utilisation, reporting a broad range of chronic and severe medical concerns and a five times higher rate of mental health service use; between 8% and 12% experienced housing insecurity due to being evicted or threatened with eviction due to hoarding (Tolin et al., 2008a, 2008b). Hoarding symptoms are associated with workplace impairment (Mathes et al., 2019), increased psychiatric co‐morbidity, childhood distress and family strain (Tolin et al., 2008a, 2008b). Hoarding is inversely related to household income (Samuels et al., 2008) and represents a community health problem involving multiple community agencies (Frost et al., 2000).

A study in the North East of England with Housing Association providers and Tyne & Wear Fire and Rescue Service found that although people who hoard comprise a relatively small sample of the population, they nevertheless present a significant economic burden to housing providers and emergency services with an estimated cost per person who hoards per year of £15,589 (Neave et al., 2017). In the same study, additional costs were found such as the 2,108 properties (of the 30,000 home checks), which presented significant fire risks due to hoarding of materials constituting a fire hazard, with 51 of these properties identified as posing a significant danger to fire officers in case of a fire. The issue of dealing with people with hoarding behaviours presents many challenges to housing providers, balancing the care of their properties with their duty of care for their tenants. When cases of serious hoarding come to the attention of a council, often through mandated building or safety inspections, there is an obligation to intervene and deal with the hoarded items due to health and safety issues. However, it is known that intervening and the removal of clutter is vastly distressing and traumatic for the individual, often leading to more severe hoarding (Muroff et al., 2011). Housing insecurity and eviction is the last, and worst option for all involved. Additionally, work in Canada points to difficulties (and the overwhelming nature of hoarding) for those who provide services around: consent for intervening, the lack of clinical management skills and training by those managing people who hoard; poor collaboration between state services, such as housing and fire services, a lack of resources for such a complex mental health problem and the need for and community partnership (Bodryzlova & O’Connor, 2018; Bodryzlova et al., 2020; Lacombe & Cossette, 2018). For people who hoard, a lack of collaboration; a lack of recognition and treatment for their complex needs and communication that is perceived as violent and threatening has the potential to worsen the clinical course of hoarding (Bodryzlova et al., 2020; Gibson, 2015).

The purpose of this research was to understand the nature, circumstances and extent that hoarding represents in one city council in England (Norwich in the east of England) with a large housing stock of over 14,500 properties. The aim was to enable the development of strategies to prevent serious hoarding, and its associated health and safety risks and understand how a housing provider can develop more holistic strategies to better support their tenants.

## 2 METHODS

We took two approaches. Firstly, to understand the nature and extent of hoarding we developed a new reporting system based on our review of the literature and our discussions with the council. For 3 months, staff in the Housing Department populated this system with information about their tenants with hoarding behaviours that they had contact with during this time. This was de‐identified and shared with the researchers. Information included the Clutter Rating Index, vulnerability of the tenant, including safeguarding referrals, children as occupants, pets, duration of tenancy with the Council, environmental health and fire risks, and a short description. We also took a qualitative approach for our work with staff in the Housing Department to understand the circumstances and context of hoarding from their point of view. To conduct this exploratory qualitative work, the research was promoted by a senior manager in the Housing Department at the council via an email designed by the researchers. Staff were requested to email the researchers for further information if they were interested in taking part. After receiving this information, they could decide not to be involved or complete an on‐line consent form. In total, 13 staff expressed interest and 11 consented to be interviewed. All interviews were conducted via Microsoft Teams, audio recorded and transcribed and all identifying information was removed. Interviews took approximately 60 min. The interview guide is appended and included asking the interviewee to talk through one of their cases to enable us to understand the complexities and circumstances of hoarding. Interviews were conducted by two experienced female post‐doctoral researchers. There was no relationship with any of the participants prior to the study. The first three interviews were independently coded by the two researchers into broad themes and subsequently there was continual discussion about the interpretation of the data and to refine our themes. The data were analysed inductively, due to this being an emerging field with little theory on the management of people who hoard, searching for patterns relevant to the questions and then themed by the two researchers using reflexive Thematic Analysis, acknowledging the highly contextual nature of the data (Braun & Clarke, 2006, 2020).

This study received institutional ethical approval from The University of East Anglia Faculty of Medicine and Health (2020/21‐144) in July 2021 including a collaboration agreement with the council for data sharing. One clear aspect of the ethical application was that staff involved would not be identifiable and representative case studies would be presented in such a way that they could not be identified.

## 3 FINDINGS

### 3.1 The city council hoarding reporting system (May to August 2021)

The trial reporting system was set up in May with information provided to the researchers in August 2021. This reported 38 tenants with indicative hoarding behaviours. Of these, 63% were male and the average age was 60 years of age (range 27–79 years). The report suggests that 47% had a known disability or vulnerability, with nearly twice as many male tenants having a reported disability (58%) than female residents (29%).

The mean duration of occupancy is 24 years (range 6–43 years). Most commonly, people with hoarding behaviours were resident in flats (58%, plus 5% residing in tower block flat accommodation) or a house or bungalow (37%). Three properties were in Sheltered housing complexes. The large majority of properties were occupied by one person (87%). There were children present in 5% of the properties and pets in 11%. According to the Clutter Image Rating Scale, the majority of the properties were rated 4–6 (47%), or 7–9 (39%) on the scale (Frost et al., 2008) (link to the scale can be found here: https://hoardingdisordersuk.org/research‐and‐resources/clutter‐image‐ratings/. There were more male residents with Clutter Rating Scale scores of 7–9 (46%) than female residents (29%). There was an Environmental Health and Fire Risk identified in 34% properties. Findings are further detailed in Table 1.

**TABLE 1:** City council hoarding report May–August 2021

	Total *n* = 38	Female *n* = 14 (37%)	Male *n* = 24 (63%)
Characteristics (*n*, %)			
Age (mean, range) (years)	60 (27–79)	58 (36–71)	62 (27–79)
Disability or vulnerability reported	18 (47%)	4 (29%)	14 (58%)
Housing information			
Occupancy duration (mean, range) (years)	24 (6–43)	25 (6–43)	24 (7–43)
Sheltered accommodation	3 (8%)	2 (14%)	1 (4%)
Property type			
House or Bungalow	14 (37%)	8 (57%)	6 (25%)
Flat	22 (58%)	6 (43%)	16 (67%)
Flat (Tower)	2 (5%)	0	2 (8%)
Single occupancy	33 (87%)	10 (71%)	23 (96%)
Child present	2 (5%)	1 (7%)	1 (4%)
Pets (1 or more)	4 (11%)	2 (14%)	2 (14%)
Hoarding and Risk			
Clutter scale rating			
1–3	5 (13%)	4 (29%)	1 (4%)
4–6	18 (47%)	6 (43%)	12 (50%)
7–9	15 (39%)	4 (29%)	11 (46%)
Environmental health risk	13 (34%)	4 (29%)	9 (37%)
Fire risk	13 (34%)	4 (29%)	9 (38%)

### 3.2 Themes from our interviews

The 11 staff interviewed had worked in housing roles (as Housing Officers, Specialist Support Officers, Tenancy Management and Public Protection) for between 2 and 20 years. They had come from a variety of backgrounds including mental health support, working with older people and environmental health. From our analysis we developed five themes: Working with others, Balancing an enforcement approach, Feeling conflicted, Complex needs of people who hoard and Staff needs, as detailed below.

#### 3.2.1 Working with others

Managing hoarding cases involved multiple agencies, and partnership working with other services (e.g., local fire department), health professionals, other council teams, as well as working with individuals and their families and friends. There were acknowledged challenges when it came to working together (difficulty making appropriate referrals (e.g., mental health), a lack of understanding across services about roles and responsibilities) and a need for a more collective approach:

*Getting that psychological support … There's a lot of gatekeeping. And that's not done out of neglect, that's done out of sheer, uhm, you know, you're talking about a finite service with almost infinite amount of people coming through the door, so getting that recognized requires quite a number of agencies to agree, and each threshold seems to be much more stringent than the next. So no, is the answer to that, which is extremely frustrating*. (p8)

*I'm dealing with a lot of substance misuse and so much mental health. I'm not an expert in this at all, but you know, it's quite clear. I think that they need some very serious intervention from mental health services. (p7)*

*And I also think that there is something really rewarding about working on hoarding cases, because you can see the changes, but you can't always do. I do, however, find them the most frustrating case that I work on because there are so many gaps in the system. And I find myself feeling very isolated on those cases*. (p8)

Hoarding was considered a ‘hot‐potato’ issue and could be bounced between services which may not consider the client was their responsibility or met the relevant criteria for need.

*I think if a person has a City Council tenancy the view would be from social services or whatever agency, you know it’s our problem … they haven't got the sort of the same pressure for finding a solution as we have 'cause obviously from our side we have a property in disrepair, possible fire risk and all that comes with it, if they have a fall*. (p7)

*often the support services that you're working with work to their own timeframes and their own criteria and when they see fit to shut a case down, and it's not up to us, is it, it's up to them, and if they've met their criteria, but there could be still outstanding issues for us, so that can be quite difficult*. (p4)There was an awareness that involving friends and families alongside the client could be either positive or problematic and required careful consideration. Staff recognised that having supportive relationships might help the client in the long term.

*Sometimes they could be really helpful and can say things and get things moving in a way that that no professional never could. And they're going to be alongside them forever, whereas we're not… But other times, it can be really destructive and actually we're looking for them to develop other more healthy, supportive relationships elsewhere that may not be their family members or their friends*. (p2)

*I have to be very careful involving family because I'm always aware of domestic violence. I'm always aware of trigger processes that family can cause. So I spend quite a long time building the relationship with the client, first understanding their view on the family and the roles that are signed within that family. Uhm and what the kind of impact of the family has been on their kind of overall life and what roles they played in that before I integrate*. (p8)

#### 3.2.2 Balancing an enforcement approach

There was a recognition from all staff of the potential harm that enforcement action (e.g., house clearances, eviction) could take and that using a holistic, person‐centred approach would have benefit. Others raised the point that having enforcement as a type of leverage was necessary, especially where it endangered others. Clearly staff saw this as a very difficult balance.

*We don't want possession, so we are really in kind of an endless cycle*. (p7)

*I think there is a bit of a responsibility to try and encourage people in all properties to make sure that their actions aren't having a detrimental effect on people who live above, below or beside them*. (p11)

*We're getting a better grasp of knowing who our hoarders are and taking a deliberate approach with them in a quite holistic way, which I think is really important*. (p2)

*We're seen as a decent landlord, we don't like to use the enforcement words, we don't like to sort of threaten things, … we're trying to help you understand that it's to be resolved before it is out of their control before it moves up the gear to become severe hoarding*. (p3)

#### 3.2.3 Feeling conflicted

It was clear from what we were told about enforcement, that staff felt very conflicted in many parts of their role. They reported a sense of conflict in what they were required to do as part of their role and a tenant's right to self‐determination and autonomy. This was particularly apparent when dealing with tenants with additional needs, such as, mental health needs, past histories of trauma and previous head injuries.

*With our role, it's split between caring for the tenant and caring for the condition of the property. Because the property is the asset and it is what you know, brings our money in but at the same time you've got to care for the tenant*. (p1)

*I would I hate it if someone came into my home and said, you know, why are you doing that? … that would upset me more than anything else, so I think I just don't ever see an end but I also think that I get a bit conflicted about whether I should really be telling people how to live their lives and that bit upsets me sometimes because I just sometimes I just want to leave them alone and let them get on with it and just think there's nothing really, they're not hurting anyone, maybe themselves*. (p4)

#### 3.2.4 The complex needs of people who hoard

All staff reported that clients required long‐term support to build relationships, especially as many tenants had previous negative experiences with the council or other statutory agencies. Additionally, cases required a long‐term approach that took account of the individual client's needs, including potential trauma.

*It's all very well doing all of that, getting that done, but they need aftercare. A lot of these people you can't leave because you know their hoarding is a mental health issue at the end of the day, you can take away and try and sort the visible part of it, but the hidden part is the hoarder themselves and what made them do that in the first place. And so, you might be tidying up the bit that everyone can see, but the actual root of the problem that caused it isn't necessarily being dealt with, is it? So, it will. It will come back*. (p10)

In addition, staff gave many examples of how they worked to engage with clients in an individualistic and compassionate way.

*You have to be so patient, it's a drip feed, build up that relationship… my first instinct is to build up a relationship, I ignore the condition of the property. I concentrate on the tenant first of all*. (p9)

Staff were all aware of the potential for underlying trauma and mental health conditions. This appeared to motivate their compassionate approach, but they also recognised the limits of their role and their expertise.

*I think of our experience of hoarding cases is there some level of trauma that's been a trigger for why they hoard. So it it's understanding what they need around. And you know, dealing with and processing that trauma. That might need to come first before we even touch on the hoarding, because there's a reason why all their stuff is there*. (p2)

*I do tend to find that a lot of people who are in you know in these situations do have learning difficulties, they've got mental health issues. They have, you know, really complex sets of needs*. (p4)

*I do work with some hoarders who you know are really receptive, but they do have really severe mental health and that's a barrier that I find because that's not my expertise. It's not my specialism, it's something that I do struggle with*. (p4)

Staff recognised the importance of developing a good relationship with their client, being non‐judgmental and consistent.

*A few of my hoarders have said this, ‘it's so nice for somebody to come around and not judge me and not have a go at me. I was so frightened to let you in because I thought I was gonna lose my tenancy’*. (p10)

Staff were also concerned about the potential extra vulnerabilities created by hoarding, for example one described two cases:

*We found asbestos that has been thrown at the bottom of the garden which potentially came from either side or the back and pushchairs and things like that thrown over* (p10)

*We do the face‐lift for them and we make it look instantly better and they can feel better about our house or prouder … that’s an important thing to focus on I think as well, what's the outside looks like.* (p10)

#### 3.2.5 Staff needs

All the staff talked through a case study, and the challenges it presented. For example, one client had no heating due to her gas supply being cut off for safety reasons and spent the winter sleeping on the sofa with portable radiators and blankets. Staff described managing these cases as tiring and frustrating with susceptibility to fatigue and burnout and impacting their resilience. There were clearly identified support and training needs for staff, and there may have been missed opportunities to help staff process their experiences of challenging cases.

*I have at times felt frightened of the extent to what I'm working with* … *You might have been the only person that's gone into that person's life for a long time as a frontline member of staff, but then you've walked away 'cause you don't have the skills or the training or the help or the tools or support to deal with it* … *I'm scared of re‐traumatizing her (case study described) by going back and talking through this stuff and actually dealing with this stuff* (p1)

*We don't know enough about what helps. I think that's why it's such a hot potato because nobody really feels confident in knowing what's the best approach*. *So we've all got some ideas that we try and use and do. But I think everybody needs to understand, you know, have a better understanding of what is helpful*. (p2)

### 3.3 Case studies

We developed six case studies from the information given to us from the 11 interviews. These can be found in the online supporting information provided to help the reader contextualise our findings and better understand the circumstances and nature of hoarding behaviours that the housing officers were working with. We were very mindful of the sensitivities and likelihood of underlying trauma relating to hoarding and therefore generalised some of the details in the case studies to ensure that individuals cannot be identified.

## 4 DISCUSSION

Hoarding is a community health and social problem with serious implications and risks for the person and their community. It represents a major challenge to housing providers and other statutory services. Our research on housing‐related issues clearly demonstrates these risks in council owned properties; for example, the particular risks for their neighbours found in flats (60% of those in our report lived in a flat), the vulnerability of the tenant (47% had a known disability or extra vulnerability due to additional mental and physical health needs) and the additional vulnerabilities that the person faced due to their hoarding behaviours. Through our interviews, and the case studies that Housing Officers provided and discussed with us at their interview, we have demonstrated some of the multifaceted needs of people who hoard and the complexity of dealing with other agencies; the challenges of enforcement strategies (harm reduction, de‐cluttering and ultimately eviction) and staff needs in this ‘frontline’ role that requires holistic person‐centred, compassionate and highly individualistic problem solving. This resonates with previous work that noted the challenges of partnership working because referrals have the potential to overwhelm services and create tensions in professional role boundaries between Housing Officers and other professionals, such as social workers (Holding et al., 2020).

The role of the Housing Officer is very complicated (beyond hoarding) and they have a key role in building coherent and inclusive neighbourhoods (Blank et al., 2021). Ultimately, working with such major challenges, Housing Departments have the responsibility to balance the needs of tenants and their right to autonomy and self‐determination (a freedom to live their lives in the way that they choose) with the responsibilities they have as a Council and public body to mitigate risk and maintain safe and habitable housing for the person who hoards, together with public safety within local communities. By its very nature, working with hoarding involves working with multidisciplinary teams and the different professions need to find common ground in effectively working together. This includes dealing with the difficult and challenging ethical dilemmas (such as resolving conflicts on rights to freedom and public safety) that are often present in hoarding cases (Koenig et al., 2010).

Our research points to the social isolation and hidden nature of hoarding. This, alongside the public stigma that people who hoard can experience, can mean that seeking help and engaging with services is a major barrier (Chasson et al., 2018). It may be that the co‐ordinated, case‐managed response developed in North America, which conceptualises hoarding as a complex community problem (rather than an individual mental health problem) and uses a supportive harm reduction approach, bringing together community organisations and networks to manage potentially dangerous hoarding behaviours (Bratiotis et al., 2019) could support the engagement needed with services. Similarly, in Vancouver a Hoarding Action Response Team (HART) works with people with severely hoarded homes in a harm reduction and case management approach to employ strategies that build relationships, set goals and coordinate services (Kysow et al., 2020). Our background work with Norwich City Council in England suggested that this is the approach that they would like to take.

Finally, The British Psychological Society's, Good Practice Guidelines on hoarding (Hare et al., 2015) recommend that everybody working with people who hoard should have access to training and information on good practice to ensure competence in the assessment of, and interventions for, hoarding. Our research sought to address their recommendation for a need for research to understand the adaptations needed by service providers to significantly improve engagement and effectiveness with individuals. There are many potential agencies that are involved and affected by hoarding behaviours, including social care staff (e.g., social workers or home‐based care providers) and voluntary organisations that work with people who hoard (Ryninks et al., 2019). There is limited research understanding the impact working with people with hoarding behaviours has on staff or volunteers (Brown & Pain, 2014; Holden et al., 2019; Ryninks et al., 2019), which would be a useful topic for future research in order to improve overall effectiveness in this area.

There are limitations to our study. The reporting system was a trial system and only recorded 38 cases as it was a new system. Our interviews, where most had a caseload of 10–12 people with hoarding behaviours, suggest that this is significant under‐reporting. Our ongoing work in partnership with the city council addresses this, and the reporting system has been promoted and is now being more extensively populated. Our interviews were offered to maintenance staff who are often the first people to report hoarding in properties, when they attempt to conduct statutory safety checks (such as gas and electric checks) or building maintenance. Unfortunately, they were too busy with work (especially since the halt to work due to Covid). Understanding this first contact with people who hoard would have been helpful for a greater understanding. The strengths of our study are that the study was co‐designed with the Council from the outset to address this, until now, neglected health and social care aspect of the housing officer role. Although a small sample, our understanding is that our sample is a fair representation of housing officers in a council.

## 5 CONCLUSIONS

The remit of modern Housing Officers encompasses a wide public health role. In confronting and dealing with issues relating to tenants with hoarding behaviours, they are a frontline workforce dealing with what are often the manifestations of complex life histories and mental health conditions. In this aspect of their role, they are balancing the rights, freedom and autonomy of an individual to live in their home in a way that they choose, with responsibilities to protect the property and the health and safety of the public. Negotiating the potential challenges of multi‐agency collaboration within such situations is a further aspect of the role for which staff need additional practical and emotional support. Our findings strongly suggest a greater focus on risk stratification and a more holistic and community‐based approach to hoarding cases to effectively deal with this most complex of social and health issues.

## Supporting information

Supplementary Material

## Data Availability

The data that support the findings of this study are available on reasonable request from the corresponding author. The data are not publicly available due to privacy and ethical restrictions.
