# Supplementary material for: Council tenancies and hoarding behaviours: A study with a large social landlord in England
Source: Health Soc Care Community. 2022 Mar 20;30(6):2292–9. doi: 10.1111/hsc.13779 (PMC10078723; doi:10.1111/hsc.13779)
Supplement: Supplementary file 1 — Supplementary Material [file HSC-30-2292-s001.docx]

**Hoarding case studies**

These cases studies are based upon the interviews with Council staff who have experience working with hoarding cases in our study. Names are pseudonyms and aspects of the cases altered to ensure that is not possible for the person to be identified.

**Hilary**

Hilary is a council tenant (aged 39) who lives on her own and has been a tenant for 15 years and lives in a flat. Hilary’s son died 4 years ago in traumatic circumstances and it was Hilary who had to identify the body. Hilary’s husband left her after the death of their son.

Hilary had collected lots of toys and they are filling up the room. Hilary has been very frightened, and this has at times arose as anger.

It took many attempts before a housing officer was allowed into the house and it was over a tea that Hilary disclosed her previous traumas. To enable checks and a safety inspection, it was agreed that the hallway would be cleared. This has been completed and the safety checks carried out.

The housing officer has not been back to the house because they are fearful that this may re-traumatize Hilary by going back and having to address the issues.

**Lionel**

Lionel is a single tenant, aged 70 years who lives in a house in the city. The case was first reported due to complaints from a neighbour because the garden is full of timber and waste. On the first visit, the property looked like there was no-one living inside, the outside of the house is in a poor condition and the housing officer does not get an answer.

After another visit, the housing officer meets Lionel who lives in the house. From an initial view, inside the house is also filled with plastic and rubbish, which Lionel explains he wants to have recycled ‘properly’. Lionel also explains that other people dump rubbish, timber etc. into the bottom of his garden, contributing to the build-up of waste.

There is a concern from environmental protection that Lionel is also at risk of vandalism or damage to the property and that the hoarding is making him vulnerable.

The housing officer is due to contact social services but is wary that they may not be keen to be involved. The officer will be visiting with the Fire Service to discuss how to manage the fire risk and aim to clear the property, with Lionel.

**Kishan**

Kishan is a tenant in a flat, aged 36 and has been a tenant for 6 years. Kishan is semi-skilled but lost his job during Covid lockdown. He has some issues with alcohol. He had found the body of a parent after they had committed suicide.

The housing officer visited as the electricity checks were overdue and found some rooms in the property filled with rubbish. Initially the housing officer found Kishan difficult to engage but after a couple visits, they were able to go into the house with the Fire Service and talk to Kishan about the fire risk and options for support.

Having returned after 1 month, the tenant had cleared a lot of the rubbish from his flat (explained that he’d had help from a friend). There is still some work to do but it is much improved. The housing officer cannot quite believe it, but it’s really pleased he has. The officer will revisit in 3 months to see if it has been refilled.

**Erica**

Erica is a 70-year-old tenant, who has been a tenant for 28 years. Erica lives with her 3 cats in a flat. There have been complaints from the neighbours about the shared access areas (hall and stairs), where items have begun to spill out.

Erica has begun to clear the living room, but there are still a large number of items (some financially valuable) in the property. The windows and doors are in urgent need of replacing as they are rotten and both the kitchen and bathroom upgrades have been missed.

Erica was visibly quite upset when the visit was made. Previously, Erica’s property had been cleared (due to Environmental Health warrant) and this left Erica distressed and is struggling to re-establish trust with housing officers.

There remains a serious concern about Fire Risk and the housing officer will visit with the local fire soon.

**Cyril**

Cyril is 71 and lives in a one-bedroom flat. Cyril has previously been diagnosed with a head injury and has generally poor physical and mental health but still gets out and about around the city. Cyril sleeps on the floor, by the front door of his flat and has no heating or hot water (the boiler has been condemned).

There are four ovens inside the kitchen that do not work, and Cyril uses a hotplate to heat his food. The upgrade to the kitchen and bathroom is overdue, plus the flat needs re-wiring, new doors and windows. Cyril attends a place in the city for homeless people to shower.

The council have been working with Cyril for over 7 years. A previous clearance removed 6.5 tonnes of material, including newspapers dating back 15 years. The flat has now returned to its previous state.

Social Worker is now involved and there are now discussions about his mental capacity, currently the way he lives is considered his choice, however this may mean that the decisions need to be taken in his best interest.

**Ameillia**

Ameillia is 63 and living in a flat, she has very poor mobility and no heating because of the hoarding (the gas had to be capped off). Ameillia had an awful winter, the council provided radiators and blankets and she was sleeping on the sofa. During this time Ameillia was not eating properly because she could not access the kitchen and there was food in the living room.

Ameillia was happy to move and a move to sheltered housing helped her choose items to take. It took six months for a place to become available and there were weekly visits before the move to enable this (phone calls each week during lockdown). Mentally, she was ready to move.

Ameillia said that her collecting started when her daughter left home and she was alone, feeling abandoned and she bought little trinkets to fill the void. She has also had a difficult relationship with ex-husband and her father. She has agreed to go to OCD support group with the Housing Support officer with her for support.

In ongoing visits to the property, there is still a lot of hoarding, but the walkways are clear and kitchen clean. She now has contact with daughter and grandchild who visits on their way home from school (previously it was unsafe in the flat). She looks better and the move has had a really positive impact on her physical and mental health.
